# Correction: Scimeca et al. Microcalcifications Drive Breast Cancer Occurrence and Development by Macrophage-Mediated Epithelial to Mesenchymal Transition. *Int. J. Mol. Sci.* **2019**, *20*, 5633

**DOI:** 10.3390/ijms25158016

**Published:** 2024-07-23

**Authors:** Manuel Scimeca, Rita Bonfiglio, Erika Menichini, Loredana Albonici, Nicoletta Urbano, Maria Teresa De Caro, Alessandro Mauriello, Orazio Schillaci, Alessandra Gambacurta, Elena Bonanno

**Affiliations:** 1 Department of Biomedicine and Prevention, University of Rome “Tor Vergata”, Via Montpellier 1, 00133 Rome, Italy; manuel.scimeca@uniroma2.it (M.S.); marydecaro12@gmail.com (M.T.D.C.); orazio.schillaci@uniroma2.it (O.S.); 2 San Raffaele University, Via di Val Cannuta 247, 00166 Rome, Italy; 3 Fondazione Umberto Veronesi (FUV), Piazza Velasca 5, 20122 Milan, Italy; 4 Saint Camillus International University of Health Sciences, Via di Sant’Alessandro, 8, 00131 Rome, Italy; 5 Department of Experimental Medicine, University “Tor Vergata”, Via Montpellier 1, 00133 Rome, Italy; bonfiglio.rita@gmail.com (R.B.); erika.menichini@gmail.com (E.M.); alessandro.mauriello@uniroma2.it (A.M.); gambacur@uniroma2.it (A.G.); 6 Department of Clinical Sciences and Translational Medicine, University of Rome “Tor Vergata”, 00133 Rome, Italy; albonici@med.uniroma2.it; 7 Nuclear Medicine, Policlinico “Tor Vergata”, 00133 Rome, Italy; n.urbano@virgilio.it; 8 Istituto di Ricovero e Cura a Carattere Scientifico (IRCCS) Neuromed, 86077 Pozzilli, Italy; 9 “Diagnostica Medica” and “Villa dei Platani”, 83100 Avellino, Italy

## Error in Figure

In the original publication [1], there was a mistake in Figure 6 as published. During the manuscript preparation, panel I of Figure 6 was erroneously uploaded. The corrected Figure 6 appears below.

**Figure 6:**
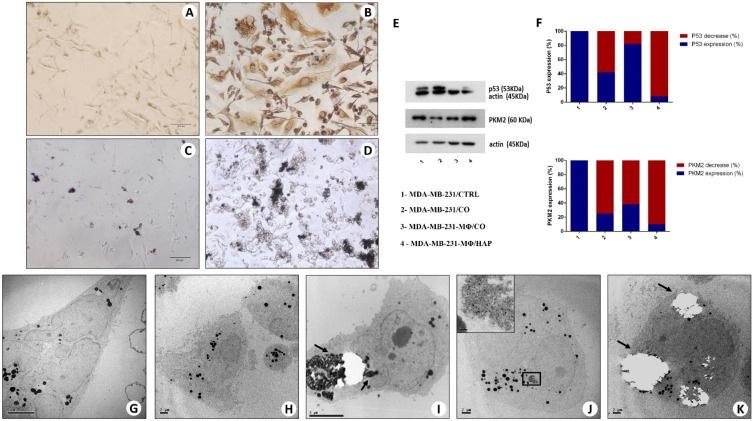
In vitro model of BOLC development. (**A**) Immunocytochemical analysis of MDA-MB-231 alone (MDA-MB-231/CTRL) shows no/few vimentin-positive cells. (**B**) Several vimentin-positive MDA-MB-231 cells are present after co-culture with CO and activated monocytes. (**C**) The image displays the starting point of MDA-MB-231 cells incubated with CO and activated monocytes. (**D**) After 10 days of culture, several large, calcified nodules are present. (**A**–**D**) scale bars represent 100 µm. (**E**) Western blot analysis for tumor protein 53 (p53) and pyruvate kinase muscle (PKM) 2. (**F**) Western blot densitometric analysis (%) of p53 and PKM2 expression compared with the respective controls (MDA). (**G**,**H**) The ultrastructural aspect of MDA-MB-231 (CTRL). (**I**–**K**) Breast osteoblast-like cells with hydroxyapatite intra-cytoplasmatic electron-dense granules (arrows) in MDA-MB-231 + Co +-activated macrophages.

Moreover, the Editorial Board requested that the authors modify Figure 5 to include representative images of fibroadenoma areas with electron micrographs that better illustrate the observations made. The corrected Figure 5 appears below.

**Figure 5:**
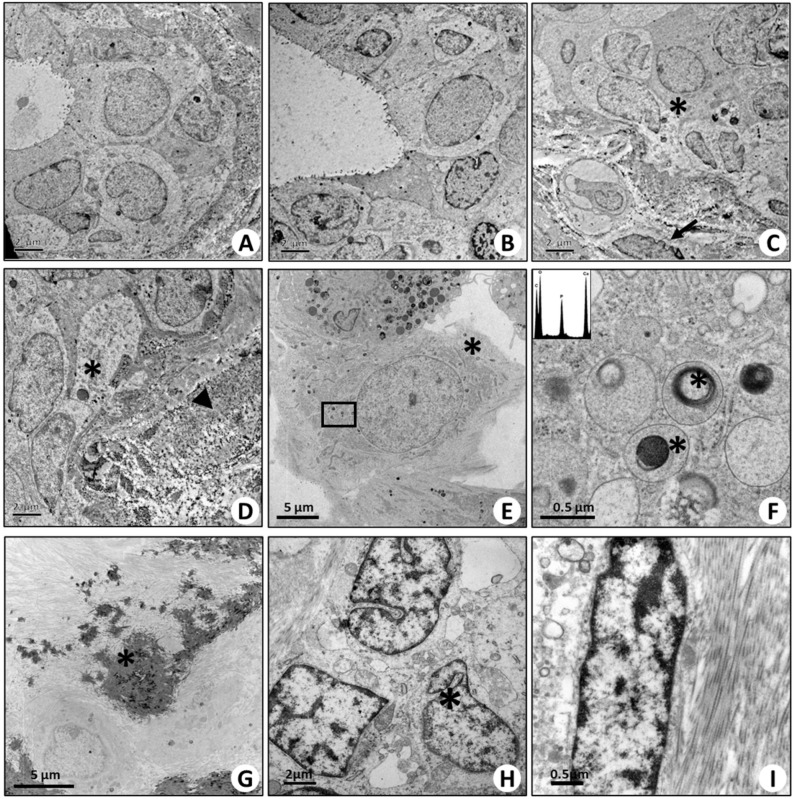
Transmission electron microscopy of breast tissues. (**A**,**B**) Images show the ultrastructure of normal ducts in adjacent areas of fibroadenoma lesions. Both myoepithelial and luminal cells are displayed. (**C**,**D**) Fibroadenoma regions are characterized by epithelial breast cells (arrows), spindle cells (asterisks), and abundant stroma (arrow heads). (**E**) Infiltrating breast cancer is characterized by the presence of microcalcifications, and vimentin-, RANKL-, OPN-, BMP-2-, BMP-4-, and PTX3-positive cancer cells show several osteoblast-like cells. Asterisks mark a cell rich in endoplasmic reticulum, with a large nucleus and some electron-dense granules. Arrows mark a large cell with numerous electron-dense granules. (**F**) High magnification of panel E. The image displays electron-dense granules containing hydroxyapatite (asterisks) (energy-dispersive X-ray (Edx) spectrum). (**G**) Calcified nodule (asterisk) in breast infiltrating carcinoma. (**H**) Several cells surrounding the calcified nodule show large nuclei (asterisks) and collagen fibers both in intracellular and extracellular space. (**I**) High-magnification image of a cell next to the calcified nodule in panel (**G**). The electron micrograph shows the presence of fiber collagen in the cytoplasm of this cell.

The authors apologize for any inconvenience caused and state that the scientific conclusions are unaffected. This correction was approved by the Academic Editor. The original publication has also been updated.
